# Effects of the *kdr* resistance mutation on the susceptibility of wild *Anopheles gambiae* populations to *Plasmodium falciparum*: a hindrance for vector control

**DOI:** 10.1186/1475-2875-13-340

**Published:** 2014-08-30

**Authors:** Mamadou Ousmane Ndiath, Aurélie Cailleau, Seynabou Mocote Diedhiou, Abdoulaye Gaye, Christian Boudin, Vincent Richard, Jean-François Trape

**Affiliations:** G4 Group, Institut Pasteur International Network, Institut Pasteur de Bangui, Bangui, BP 923 Central African Republic; Afrique One, Centre Suisse de recherché Scientifiques, Abidjan, 01 BP 1303 Côte d’Ivoire; Departement of Animal Biology, Cheikh Anta Diop University, Dakar, Fann, LEVP PO Box 5005, Sénégal; Medical Research Concil Unit The Gambia Atlantic Boulevard, Banjul, Fajara, PO Box 273, The Gambia; Laboratoire de Paludologie et de Zoologie Médicale, Campus UCAD-IRD, Dakar, BP 1386 The Gambia; Unité Epidémiologie, Institut Pasteur de Dakar, Dakar, Sénégal

**Keywords:** *Anopheles*, Susceptibility, Infection, *kdr* resistance, Dielmo, Senegal

## Abstract

**Background:**

In the context of generalization of insecticide resistance, the hypothesis that insecticide resistance has a positive impact on the capacity of mosquitoes to transmit malaria constitutes a hindrance for malaria elimination. The aim of this study was to investigated populations of *Anopheles coluzzii* and *Anopheles gambiae* S molecular form to assess whether different genotypes at the *kdr* locus are responsible for different susceptibility to *Plasmodium falciparum* infection.

**Methods:**

F3 progeny of *An. gambiae s.l.* collected in Dielmo were infected by direct membrane feeding with *P. falciparum* gametocyte-containing blood sampled from volunteer patients. The presence of oocysts was determined by light microscopy after seven days, and the presence of sporozoites by ELISA after 14 days. Mosquito species and molecular forms were identified by PCR. Generalized linear models were performed using the R software to test the effect of explanatory variables including the genotype at the *kdr* locus on infection rate and density.

**Results:**

The odds of being infected with oocysts and sporozoites were greater in RS and RR groups than in SS groups (χ^2^ = 42.8, df = 1, P(>χ^2^) = 6.1e-11). The density of infection was also dependent on genotype, with RR and RS genotypes showing denser infection than SS genotypes. Pairwise comparisons of oocyst number and absorbance indicated sometime a small betwen species (i.e. between *An. gambiae* S form, and *An. coluzzii*), but the effect of genotype was much more important.

**Conclusion:**

The presence of the resistance allele at the *kdr* locus increases susceptibility to *Plasmodium* not only at the oocyst stage but also at the sporozoite stage in non-genetically modified wild mosquitoes. These results have significant implications and should be taken into account in the development of strategies for malaria control.

## Background

Despite much work in basic and applied research, malaria remains, 120 years after the identification of *Plasmodium*, a major health issue, particularly in Africa [1–3]. Vector control is an important component of malaria control, and insecticide-treated nets (ITNs) and indoor residual spraying (IRS) are the front-line tools [4, 5]. Currently, pyrethroids are the only class of insecticides approved for treating bed nets because of their effectiveness, with a strong excito-repellent effect on mosquitoes, and their lower mammalian toxicity than organochlorine, carbamate and organophosphate compounds [6]. Unfortunately, a gene-conferring resistance (knock-down resistance, *kdr*) to pyrethroids and cross-resistance to DDT, first reported in *Anopheles gambiae s.s.* populations in Côte d’Ivoire [7], has spread, mainly in West Africa. *kdr*, resulting from a single point mutation was probably due first to intensive use of DDT and then pyrethroids for crop protection, particularly in cotton-growing areas and at lower rates for domestic protection [8]. The efficacy of ITNs for preventing malaria is well established and they are known to provide substantial protection to both individuals and communities using them [9]. Several studies have shown a direct relationship between the rapid increase in the frequency of *kdr* and widespread use of bed nets, with a rebound of malaria as a direct consequence [10–12].

In West Africa, the principal malaria vectors are members of the *An. gambiae* complex [13]. Over the past 15 years, several research teams have agreed on a molecular approach to speciate *An. gambiae s.s.* Five sympatric and syntopic chromosomal forms of *An. gambiae s.s.* have been described and designated with non-Linnean nomenclature as bamako, bissau, forest, mopti and savanna [13–15]. The pattern of molecular markers revealed the existence of two genetic variants, referred to as molecular M and S forms [16–18]. No association was found between speciation and chromosomal constitution, which seems to be involved in ecotypic adaptation. Although chromosome inversions and even chromosome-2 karyotypes are shared between molecular forms, there is a significant lack of gene flow between the M and S forms because of the absence or rarity of hybrid rDNA genotypes [14, 19, 20], as seen in geographically comprehensive surveys in Africa [17, 18, 21–23]. Whatever the geographical region, however, gene flow between the M and S forms is very limited, resulting in the current speciation. On the basis of the investigation of Reidenbach *et al*. [24] on the genomes of paired population samples of M and S from Burkina Faso, Cameroon and Mali, Coetzee *et al.* [25] assigned the name *An. gambiae* to the S molecular form and *Anopheles coluzzii* to the M form.

*Plasmodium* species, the agents of malaria, are exclusively transmitted by *Anopheles* mosquitoes. The susceptibility of these mosquitoes to *Plasmodium* infection is related to their ability to allow parasite development from gamete fertilization through to sporozoite production. During sporogonic development in the mosquito midgut lumen, midgut epithelium and haemolymph, the parasites face a hostile environment, leading to a considerable reduction in the number that reach the oocyst stage [26–28]. Mosquito susceptibility is the result of evolutionary processes in both the parasite and the vector, which maintain susceptible and refractory alleles in natural populations. Susceptibility is highly variable, ranging from total refractoriness to high receptiveness depending on both parasite and vector status and their interactions [29]. In the context of generalization of insecticide resistance, the hypothesis that insecticide resistance has a positive impact on the capacity of mosquitoes to transmit malaria constitutes a hindrance for malaria elimination [30].

The aim of this study was to test whether the *kdr* mutation in wild *An. gambiae* affects its susceptibility to *Plasmodium* infection. As populations of *An. coluzzii* (previously molecular form M) and *An. gambiae* S molecular form have been shown to have different susceptibilities to *Plasmodium* [31]. These studies investigated these two populations to assess whether the genotype at the *kdr* locus is responsible for different susceptibility to *Plasmodium falciparum* infection.

## Methods

### Mosquitoes

*Anopheles gambiae s.s.* (molecular form S) and *An. coluzzii* larvae were collected at ten breeding sites in the village of Dielmo [32] (13°43’N, 16°24’W) between August and September 2012. Larvae were raised until emergence; adults were fed on rabbit blood, and 200 females (F0) were randomly selected (20 from each collection site). Each F0 female was allowed to lay its eggs individually before it was genotyped for species and molecular form by PCR-RFLP [14].

The frequency of *kdr* was determined before (in the F0 population) and after infection (in F4 population) but not in the rearing females (F1 to F3 populations), for which only the molecular form was determined. Previous studies have shown that the *kdr* frequency in *Anopheles* populations in Dielmo can reach 47% [12, 33] and may increase significantly after inter-generational crosses.

The offspring of F0 females of the same taxa were then pooled and bred under the same conditions. Larvae were fed Tetramin fish food. Pupae were collected and placed in 10-L plastic buckets, which were covered with mosquito gauze with a cotton sleeve for introducing 10% glucose on filter paper. Adults were maintained at 28°C, 80% relative humidity and 12:12 hr light:dark cycle. In order to increase the proportion of mosquitoes accustomed to feeding on membrane, aggressive F1, F2 and F3 females were selected.

F4 females used for infection were genotyped, and species and molecular forms were confirmed by PCR RFLP [14]. L1014F and L1014S *kdr* mutations (hereafter referred to as *kdr*-w and *kdr*-e, respectively) were detected by PCR [34, 35].

### Gametocyte carriers

Gametocyte carriers were detected in cross-sectional surveys in villages and schools during the high transmission period (October–November) in Anene (14°47'N, 16°55'W Thies region). Finger-prick blood was taken from each volunteer. Thick blood smears were stained with 10% Giemsa and examined microscopically under a (100×) oil immersion lens for the presence of sexual and asexual parasites. Parasite density was estimated by counting against 1,000 white blood cells and converted to numbers of parasites per microlitre by assuming a standard white blood cell count of 8,000/μL. Symptomatic and non-symptomatic individuals with asexual parasites were treated with artemisinin-based combination therapy according to national recommendations. The inclusion criteria for gametocyte carriers were: age over ten years, a *P. falciparum* gametocyte density over 20/mm^3^ of blood and no anti-malarial treatment in the previous month. Each gametocyte carrier provided 6 mL of blood drawn into a heparinized vacutainer tube, and each was given an insecticide-impregnated bed net in compensation.

### Ethical approval

Experiments involving human subjects, population screening and collection of blood samples were conducted in full accordance with ethical principles. Free and informed consent of the donors or their guardians was obtained at all times, and community consent was obtained beforehand. Regular audits were conducted by the National Ethics Committee of Senegal and *ad hoc* committees of the Ministry of Health. This study was approved by the Ethical National Comittee of Senegal.

### Direct membrane feeding assay

Experimental infections were carried out in the direct membrane feeding assay as described by Mulder *et al.* [36]. Blood was rapidly distributed to two pools of three-day-old females of each taxon through a serially connected, warm water (37°C), jacketed membrane feeder, and the mosquitoes were allowed to feed for 15 min; then, partially fed and non-fed specimens were removed. Two batches of 50 mosquitoes of each taxon were randomly selected from among fed females and maintained in the insectary on a 10% sucrose diet for further analyses. The first batch of mosquitoes was dissected seven days later, and their midguts were stained with 3% mercurochrome in PBS and examined under a light microscope (40× objective) for detection and quantification of oocysts. The presence or absence of oocysts (status of infection by oocyst) and their number (intensity of infection) were recorded. The heads and thoraxes of the second batch of mosquitoes were used 14 days after feeding to evaluate the presence of the circumsporozoite protein of *P. falciparum* in an enzyme-linked immunosorbent assay (ELISA) [37]. A mosquito was considered to have sporozoites when the optical density was >0.6, which is that of the control strain. The status of infection by sporozoites and the optical density (proxy for the intensity of infection) were recorded. PCR RFLP [14] was performed on the carcasses of dissected mosquitoes, and the identity of the molecular forms was confirmed.

Experiments were repeated five times on different days with different samples of gametocyte-containing blood. Gametocytaemia was 78, 92, 113, 136, and 218 gametocytes/μL in the five assays.

### Statistical analysis

Susceptibility to oocysts and sporozoites were assessed separately. The first batch of mosquitoes was used to study infection by oocysts (N = 445, 358 infected), while the second batch was used to study infection by sporozoites (N = 303, 244 infected).

Susceptibility to *Plasmodium* was measured in both datasets with two response variables. The status of infection (0/1) was the variable of main interest, while the density of parasites in infected individuals (number of oocysts for the first batch of mosquitoes and absorbance as a proxy for the second batch) was used to perform a secondary, exploratory analysis.

The fixed effects of strain (*An. coluzzii* and *An. gambiae* S form), genotype at the *kdr* locus (RR, RS and SS) and the random effect of assay (a five-level categorical control variable accounting for the day of dissection and the donor) were tested.

Statistical analyses were performed with R software v3.0.2 . The overall method was the same for all four analyses (i.e., of each of the two responses variables in each of the two datasets). First, a model containing all explanatory variables and the strain-genotype interaction was adjusted with a linear mixed-effect model. The *glm* (fixed-effect generalized linear model) and *glmer* (mixed generalized linear model) procedures with binomial error distribution were used to analyse the status of infection, the *glm* and *glmer* procedures with negative binomial error distribution to analyse oocyst numbers in infected individuals, and the *lm* (fixed-effect linear model) and *lmer* (mixed linear model) procedures with Gaussian error distribution to analyse sporozoite density in infected individuals (because absorbance exhibited a Gaussian distribution). Secondly, the significance of the assay effect was assessed. Fixed-effect and mixed models where compared with the Akaike information criterion (AIC): the model with the lowest AIC was chosen. Thirdly, the best model was selected step by step with the *drop1* procedure, which performs a Chi-square test for linear models and a likelihood ratio test (approximating a Chi-square distribution) for generalized linear models. If a variable was not significant, it was removed from the model. A *p* value of ≤0.05 was considered significant.

To analyse infection rates, odds ratios (ORs) were obtained from the model estimates, which are logarithms of ORs (OR = exp^estimates^), and their 95% confidence intervals (CIs) were computed with the *confint* procedure. For oocyst density analysis, the number of oocysts in a mosquito when infection occurred was calculated from the model estimates. For sporozoite density analysis, estimates are meaningless, as the absorbance is not expected to vary linearly with sporozoite density, therefore the focus will be put on variables significativity and the trend given by these estimates.

Pairwise comparisons were then done to determine differences with the *difflsmeans* procedure (*lmerTest* library, applicable to mixed-effect models), the *wald.test* procedure (*aod* library, applicable to *glm*) and individual Welsh-corrected *t*-tests with the Bonferonni correction (linear model).

## Results

### Infection rate

The strain-genotype interaction, the strain and the assay effects were not significant in either the oocyst model nor the sporozoite infection model, while the effect of genotype (SS, RS and RR) was significant (Table 1). Therefore this is the output of fixed-effect models where only the genotype variable is kept as an explanatory variable that is presented.

**Table 1:** **Interaction of oocyst an sporozoite parameters (infection rate, oocyst number and absorbance) between assay, genotype (RR, RS and SS) and genotype-strain**

Analysis	Variable		Df	Statistic	***p***
Oocyst infection rate	Assay		4	LRT = 0.51	0.97
	Genotype-strain		2	χ^2^ = 0.26	0.88
	Genotype		2	χ^2^ = 177.9	**< 2.2e-16**
	Strain		1	χ^2^ = 2.7	0.1
Sporozoite infection rate	Assay		4	χ^2^ = 0.94	0.92
	Genotype-strain		2	χ^2^ = 1.9	0.39
	Genotype		2	χ^2^ = 151.7	**< 2.2e-16**
	Strain		1	χ^2^ = 1.4	0.23
Oocyst number	Assay (random)	fem	6	AIC = 2,782	-
		mm	8	AIC = 2,668	
	Genotype-strain		2	χ^2^ = 21.26	**2.415e-05**
	Genotype		No test needed since the interaction is significant		
	Strain				
Absorbance (Sporozoite density)	Assay (random)	fem	8	AIC = -141	-
		mm	7	AIC = -157	
	Genotype-strain		2	χ^2^ = 0.82	0.66
	Genotype		2	χ^2^ = 81.5	**3.291e-16**
	Strain		1	χ^2^ = 11.3	**0.0008**

Random variables’ significance was evaluated by comparing the Akaike information criterion (AIC) of the most complex model, which included the random effect (mm, mixed model), and that of the same model with the random effect removed (fem, fixed-effect model). The model with the lowest AIC was chosen, i.e., the random effect was kept if the model in which it was included had the lowest AIC. The significance of fixed-effect variables was tested in a Chi-square test in the linear model of absorbance or a likelihood ratio test (which assumes a Chi-square distribution) in *glm* (generalized linear model), i.e., the three other analyses. The fixed-effect variable was considered significant and kept in the model if *p* <0.05 (in bold).

**Figure 1:**
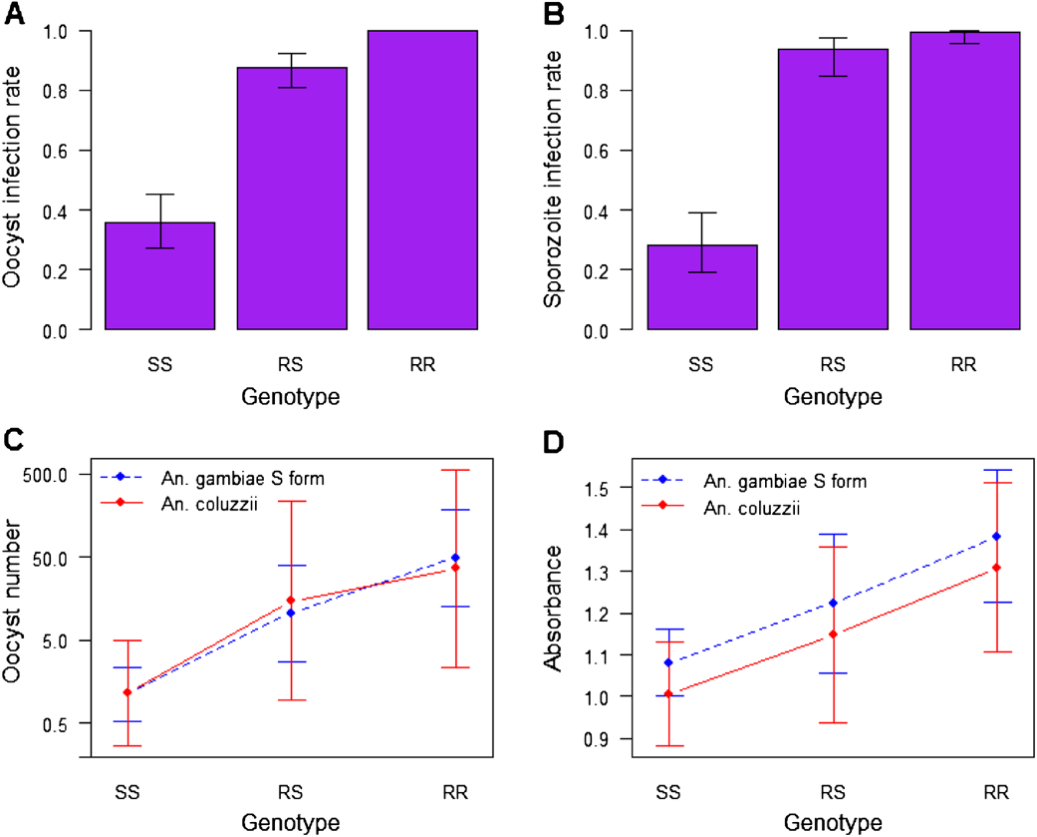
**Graphical representations of (A) oocyst infection rate, (B) sporozoite infection rate, (C) number of oocyst per midgut and (D) absorbance as a proxy for sporozoite density, as predicted with their significant explanatory variables.** Bars represent 95% confidence intervals. In C, as assay was significant in the oocyst number model, the medium assay (gametocytaemia = 113 gametocytes/μL) was chosen for the graphical representation. The intercept changed slightly for the four other assays.

The odds of being infected with oocysts were signicantly greater in RS than in SS groups (χ^2^ = 42.8, df = 1, P(>χ^2^) = 6.1e-11). As all RR individuals were infected (no variability), it was not possble to test how significantly different these individuals are from others, but it appears that homozygote-resistant individuals are much more sensitive to oocyst infection than SS (and probably RS) individuals (Figure 1A).

The results for sporozoite infection were qualitatively similar. The odds of being infected were greater in RS (χ^2^ = 39.8, df = 1, P > χ^2^ = 2.8e-10) and RR (χ^2^ = 38.4, df = 1, P > χ^2^ = 5.8e-10) than in SS genotypes (Figure 1B). The odds of being infected were also significantly higher in RR than in RS groups (χ^2^ = 4.5, df = 1, P > χ^2^ = 0.034.

### Intensity of infection

In the oocyst number model, assay, strain-genotype interaction, strain and genotype were all significant. Hence a mixed-model involving all these variableswas used to test significances and calculate estimates. In the model of sporozoite density (approximated by absorbance), the effects of assay and strain-genotype interaction were not significant, but the effects of strain and genotype were significant. Hence a fixed-effect model involving significances variables was used to test significances and calculate estimates (Table 1).

Pairwise comparisons of oocyst number and absorbance (Tables 2 and 3) indicated a small effect of species (small differences in oocyst numbers, barely significant *p* values) but a strong effect of genotype (larger differences in oocyst numbers, highly significant *p* values). Thus, the effect of species on the intensity of infection is much smaller than that of genotype.

**Table 2:** **Genotypes (RR, RS and SS) and species (An. gambiae and An. coluzzii) comparisons for infection by oocysts**

Group	Category 1	Category 2	Difference C1–C2	T	df	***p***
***An. gambiae***	SS	RS	-2.2	-2.7643	177,837.2	**< 2e-16**
	SS	RS	-3.8	-4.2698	177,885.9	**< 2e-16**
	RS	RR	-1.5	-1.8111	177,868.5	**< 2e-16**
***An. coluzzii***	SS	RS	-2.6	-2.9996	177,863.4	**< 2e-16**
	SS	RS	-3.5	-3.8752	178,162.7	**< 2e-16**
	RS	RR	-0.9	-1.2173	178,173.7	**< 2e-16**
**SS**	*An. gambiae*	*An. coluzzii*	0.0	-0.03	177,784.3	0.98
**RS**	*An. gambiae*	*An. coluzzii*	-0.3	-2.00	177,699.3	**0.05**
**RR**	*An. gambiae*	*An. coluzzii*	0.3	2.17	177,743.0	**0.03**

Further, a test was done to see whether these estimates were significantly different from zero, i.e., whether the two genotypes are significantly different, with the *difflsmeans* procedure. *p* ≤0.05 was considered significant (in bold). Note that between-species differences are lower (and barely significant) than between-genotype differences.

**Table 3:** **Genotypes (RR, RS and SS) and species (** ***An*** **.** ***gambiae*** **and** ***An*** **.** ***coluzzii*** **) comparisonsfor sporozoite density**

Group	Category 1	Category 2	t	df	***p***
***An. gambiae***	SS	RS	-3.2248	6.337	0.017
	SS	RR	-6.125	6.254	**7.3e-04**
	RS	RR	-6.1596	93.31	**1.8e-08**
***An. coluzzii***	SS	RS	-3.1699	33.56	**0.0032**
	SS	RR	-7.2688	34.57	**1.8e-08**
	RS	RR	-4.1776	65.305	**8.9e-05**
**SS**	*An. gambiae*	*An. coluzzii*	0.1201	8.171	0.90
**RS**	*An. gambiae*	*An. coluzzii*	2.0235	48.098	0.049
**RR**	*An. gambiae*	*An. coluzzii*	2.7846	144.865	0.006

Differences between genotypes and species were tested with individual Welsh-corrected two-sample *t* tests. *p* ≤0.0056 was considered significant (lowered to account for multiple testing; in bold).

More specifically, oocyst density is significantly more important in RS and RR than in SS, and significantly more important in RR than in RS. This is true both for *An. gambiae* S form and *An. coluzzii* (Figure 1C, Table 2).

Sporozoite density was greater in RS and RR than in SS individuals and greater in *An. gambiae* S form than in *An. coluzzii* individuals (Figure 1D). Pairwise comparison indicated that differences are all significant, e.g., there is both an effect of genotype and species on sporozoite density (Table 3).

## Discussion

This study is the first to evaluate the effects of the *kdr* resistance mutation on the susceptibility of a natural *Anopheles gambiae* populations to *Plasmodium falciparum*. These results showed no difference in the susceptibility of the S form of *An. gambiae* and *An. coluzzii*, corroborating those in Burkina Faso by Gneme *et al.* [38], who found equivalent susceptibility in the S form of *An. gambiae, An. coluzzii* and *Anopheles arabiensis*. In this study, however, mosquitoes with the RR genotype were more susceptible than RS mosquitoes, which in turn were more susceptible than mosquitoes without the *kdr* resistance gene, confirming the results of Alout *et al.* in Burkina Faso [39].

Results on the intensity of infection show differences between species and between genotype with a stronger effect of genotype. Species differences have already been reported [31]. The results show that mosquitoes with a resistant allele at the *kdr* locus (RR and RS genotypes) are more susceptible to parasite infection than those with the SS genotype. Alout *et al.* [39] reported conflicting results for infection rates and infection intensity, and found an increased rate of *kdr* mutation but a lower infection intensity in resistant than in susceptible mosquitoes. Methodological bias may account for this contradiction; for example, maintaining a strain for several generations (‘intra-generational fitness’) [40] and then making genetic changes by introgression might have played a role in the drastic decline observed by Alout *et al.* [39]. Furthermore, conducting a study on just one species of the *An. gambiae* complex is restrictive, as most of the species that transmit malaria belong to complexes [29, 41]. In addition, several studies have shown that various constraints (physical, physiological, immunological) during development of the parasite in *Anopheles* may significantly decrease parasite density. Such decreases were observed at several levels. While a drastic decline in parasite production is observed, especially between the parasitic gametocyte and oocyst stages, it may also occur, under certain conditions, between the oocyst and sporozoite stages [27, 28, 42].

Other resistance mechanisms than *kdr* resistance [11], such as biochemical resistance, might directly or indirectly affect activation of the immune system of mosquitoes, leading to wide variations in the parasite during its development [43]. Several genes may be implicated in the anti-*Plasmodium* immune response, including defensin and cecropin [44, 45]. A combined effect of these genes and resistance genes on the immune response might modify the effect of resistance genes altought it has not yet been demonstrated [46]. More investigations are therefore required to test this hypothesis.

It might be assumed that the presence of a resistant allele at the *kdr* locus has a significant effect on anopheline longevity, resulting in a higher rate of infection in populations that carry the gene. In this study, however, mosquitoes had the opportunity to be infected only once, so that the rate of infection is not linked to longevity but to lower immune competence. This could directly affect vector control, because the risk for malaria transmission could be greater than before introduction of bed nets for people not using bed nets, if the nets are removed, or if they lose enough of their effectiveness. Nevertheless, if the R allele decreases immune refractoriness to *Plasmodium*, it might also reduce that to other pathogens infecting *Anopheles*. Should this be confirmed, use of fungi and other parasites that infect and kill *Anopheles* could become the tool of choice in malaria control.

## Conclusion

This study is the first of its kind to show that the presence of the allele of resistance at the *kdr* locus increases the susceptibility of *Anopheles* to *Plasmodium*, not only at the oocyst stage but also at the sporozoite stage, in non-genetically modified wild mosquitoes. These results have significant implications and should be taken into account in the development of strategies for malaria control.

## References

[1] WHO (2011). World Malaria Report 2011.

[2] Greenwood B (2008). Progress in malaria control in endemic areas. Travel Med Infect Dis.

[3] Gething PW, Patil AP, Smith DL, Guerra CA, Elyazar IR, Johnston GL, Tatem AJ, Hay SI (2011). A new world malaria map: *Plasmodium falciparum* endemicity in 2010. Malar J.

[4] Feachem R, Sabot O (2008). A new global malaria eradication strategy. Lancet.

[5] Greenwood B (2009). Can malaria be eliminated?. Trans R Soc Trop Med Hyg.

[6] Curtis CF (1994). Should DDT continue to be recommended for malaria vector control?. Med Vet Entomol.

[7] Chandre F, Darriet F, Manguin S, Brengues C, Carnevale P, Guillet P (1999). Pyrethroid cross resistance spectrum among populations of *Anopheles gambiae s.s.* from Cote d'Ivoire. J Am Mosq Control Assoc.

[8] Diabate A, Baldet T, Chandre F, Akoobeto M, Guiguemde TR, Darriet F, Brengues C, Guillet P, Hemingway J, Small GJ, Hougard JM (2002). The role of agricultural use of insecticides in resistance to pyrethroids in *Anopheles gambiae s.l.* in Burkina Faso. Am J Trop Med Hyg.

[9] Takken W, Knols BG (2009). Malaria vector control: current and future strategies. Trends Parasitol.

[10] Corbel V, Akogbeto M, Damien GB, Djenontin A, Chandre F, Rogier C, Moiroux N, Chabi J, Banganna B, Padonou GG, Henry MC (2012). Combination of malaria vector control interventions in pyrethroid resistance area in Benin: a cluster randomised controlled trial. Lancet Infect Dis.

[11] Sokhna C, Ndiath MO, Rogier C (2013). The changes in mosquito vector behaviour and the emerging resistance to insecticides will challenge the decline of malaria. Clin Microbiol Infect.

[12] Trape JF, Tall A, Diagne N, Ndiath O, Ly AB, Faye J, Dieye-Ba F, Roucher C, Bouganali C, Badiane A, Sarr FD, Mazenot C, Touré-Baldé A, Raoult D, Druilhe P, Mercereau-Puijalon O, Rogier C, Sokhna C (2011). Malaria morbidity and pyrethroid resistance after the introduction of insecticide-treated bednets and artemisinin-based combination therapies: a longitudinal study. Lancet Infect Dis.

[13] Coluzzi M, Sabatini A, Della Torre A, Di Deco MA, Petrarca V (2002). A polytene chromosome analysis of the *Anopheles gambiae* species complex. Science.

[14] Favia G, Dimopoulos G, Della Torre A, Toure YT, Coluzzi M, Louis C (1994). Polymorphisms detected by random PCR distinguish between different chromosomal forms of *Anopheles gambiae*. Proc Natl Acad Sci U S A.

[15] Toure YT, Petrarca V, Traore SF, Coulibaly A, Maiga HM, Sankare O, Sow M, Di Deco MA, Coluzzi M (1998). The distribution and inversion polymorphism of chromosomally recognized taxa of the *Anopheles gambiae* complex in Mali, West Africa. Parassitologia.

[16] Della Torre A, Fanello C, Akogbeto M, Dossou-yovo J, Favia G, Petrarca V, Coluzzi M (2001). Molecular evidence of incipient speciation within *Anopheles gambiae s.s.* in West Africa. Insect Mol Biol.

[17] Della Torre A, Tu Z, Petrarca V (2005). On the distribution and genetic differentiation of *Anopheles gambiae s.s.* molecular forms. Insect Biochem Mol Biol.

[18] Wondji C, Simard F, Fontenille D (2002). Evidence for genetic differentiation between the molecular forms M and S within the Forest chromosomal form of *Anopheles gambiae* in an area of sympatry. Insect Mol Biol.

[19] Fanello C, Santolamazza F, Della Torre A (2002). Simultaneous identification of species and molecular forms of the *Anopheles gambiae* complex by PCR-RFLP. Med Vet Entomol.

[20] Favia G, Della Torre A, Bagayoko M, Lanfrancotti A, Sagnon N, Toure YT, Coluzzi M (1997). Molecular identification of sympatric chromosomal forms of *Anopheles gambiae* and further evidence of their reproductive isolation. Insect Mol Biol.

[21] Ndiath MO, Brengues C, Konate L, Sokhna C, Boudin C, Trape JF, Fontenille D (2008). Dynamics of transmission of *Plasmodium falciparum* by *Anopheles arabiensis* and the molecular forms M and S of Anopheles gambiae in Dielmo, Senegal. Malar J.

[22] Slotman MA, Tripet F, Cornel AJ, Meneses CR, Lee Y, Reimer LJ, Thiemann TC, Fondjo E, Fofana A, Traore SF, Lanzaro GC (2007). Evidence for subdivision within the M molecular form of *Anopheles gambiae*. Mol Ecol.

[23] Yawson AE, Weetman D, Wilson MD, Donnelly MJ (2007). Ecological zones rather than molecular forms predict genetic differentiation in the malaria vector Anopheles gambiae s.s. in Ghana. Genetics.

[24] Reidenbach KR, Neafsey DE, Costantini C, Sagnon N, Simard F, Ragland GJ, Egan SP, Feder JL, Muskavitch MA, Besansky NJ (2012). Patterns of genomic differentiation between ecologically differentiated M and S forms of *Anopheles gambiae* in West and Central Africa. Genome Biol Evol.

[25] Coetze M, Hunt RH, Wilkerson R, Della Torre A, Coulibaly MB, Besansky NJ (2013). *Anopheles coluzzii* and *Anopheles amharicus*, new members of the *Anopheles gambiae* complex. Zootaxa.

[26] Beier JC (1998). Malaria parasite development in mosquitoes. Annu Rev Entomol.

[27] Bonnet S, Gouagna C, Safeukui I, Meunier JY, Boudin C (2000). Comparison of artificial membrane feeding with direct skin feeding to estimate infectiousness of Plasmodium falciparum gametocyte carriers to mosquitoes. Trans R Soc Trop Med Hyg.

[28] Vaughan J, Hensley L, Beier JC (1994). Sporogonic development of *P. yoelii* in five anopheline species. J Parasitol.

[29] Cohuet A, Harris C, Robert V, Fontenille D (2010). Evolutionary forces on Anopheles: what makes a malaria vector?. Trends Parasitol.

[30] Felix RC, Muller P, Ribeiro V, Ranson H, Silveira H (2010). Plasmodium infection alters Anopheles gambiae detoxification gene expression. BMC Genomics.

[31] Ndiath MO, Cohuet A, Gaye A, Konate L, Mazenot C, Faye O, Boudin C, Sokhna C, Trape JF (2011). Comparative susceptibility to *Plasmodium falciparum* of the molecular forms M and S of *Anopheles gambiae* and *Anopheles arabiensis*. Malar J.

[32] Sougoufara S, Diedhiou SM, Doucoure S, Diagne N, Sembene PM, Harry M, Trape JF, Sokhna C, Ndiath MO (2014). Biting by *Anopheles funestus* in broad daylight after use of long-lasting insecticidal nets: a new challenge to malaria elimination. Malar J.

[33] Ndiath MO, Sougoufara S, Gaye A, Mazenot C, Konate L, Faye O, Sokhna C, Trape JF (2012). Resistance to DDT and pyrethroids and increased kdr mutation frequency in An. gambiae after the implementation of permethrin-treated nets in Senegal. PLoS One.

[34] Martinez-Torres D, Chandre F, Williamson MS, Darriet F, Berge JB, Devonshire AL, Guillet P, Pasteur N, Pauron D (1998). Molecular characterization of pyrethroid knockdown resistance (kdr) in the major malaria vector *Anopheles gambiae s.s*. Insect Mol Biol.

[35] Ranson H, Jensen B, Vulule JM, Wang X, Hemingway J, Collins FH (2000). Identification of a point mutation in the voltage-gated sodium channel gene of Kenyan *Anopheles gambiae* associated with resistance to DDT and pyrethroids. Insect Mol Biol.

[36] Mulder B, Lensen T, Tchuinkam T, Roeffen W, Verhave JP, Boudin C, Sauerwein R (1999). *Plasmodium falciparum*: membrane feeding assays and competition ELISAs for the measurement of transmission reduction in sera from Cameroon. Exp Parasitol.

[37] Beier JC, Copeland RS, Onyango FK, Asiago CM, Ramadhan M, Koech DK, Roberts CR (1991). Plasmodium species identification by ELISA for sporozoites removed from dried dissection slides. J Med Entomol.

[38] Gneme A, Guelbeogo WM, Riehle MM, Sanou A, Traore A, Zongo S, Eiglmeier K, Kabre GB, Sagnon N, Vernick KD (2013). Equivalent susceptibility of *Anopheles gambiae* M and S molecular forms and *Anopheles arabiensis* to *Plasmodium falciparum* infection in Burkina Faso. Malar J.

[39] Alout H, Ndam NT, Sandeu MM, Djegbe I, Chandre F, Dabire RK, Djogbenou LS, Corbel V, Cohuet A (2013). Insecticide resistance alleles affect vector competence of Anopheles gambiae s.s. for Plasmodium falciparum field isolates. PLoS One.

[40] Norris DE, Shurtleff AC, Toure YT, Lanzaro GC (2001). Microsatellite DNA polymorphism and heterozygosity among field and laboratory populations of *Anopheles gambiae* s.s. (Diptera: Culicidae). J Med Entomol.

[41] Coluzzi M, Sabatini A, Petrarca V, Di Deco MA (1979). Chromosomal differentiation and adaptation to human environments in the *Anopheles gambiae* complex. Trans R Soc Trop Med Hyg.

[42] Villalon JM, Ghosh A, Jacobs-Lorena M (2003). The peritrophic matrix limits the rate of digestion in adult *Anopheles stephensi* and *Aedes aegypti* mosquitoes. J Insect Physiol.

[43] Rivero A, Vezilier J, Weill M, Read AF, Gandon S (2010). Insecticide control of vector-borne diseases: when is insecticide resistance a problem?. PLoS Pathog.

[44] Dimopoulos G, Richman A, Muller HM, Kafatos FC (1997). Molecular immune responses of the mosquito *Anopheles gambiae* to bacteria and malaria parasites. Proc Natl Acad Sci U S A.

[45] Vizioli J, Bulet P, Charlet M, Lowenberger C, Blass C, Muller HM, Dimopoulos G, Hoffmann J, Kafatos FC, Richman A (2000). Cloning and analysis of a cecropin gene from the malaria vector mosquito, *Anopheles gambiae*. Insect Mol Biol.

[46] Vontas J, Blass C, Koutsos AC, David JP, Kafatos FC, Louis C, Hemingway J, Christophides GK, Ranson H (2005). Gene expression in insecticide resistant and susceptible *Anopheles gambiae* strains constitutively or after insecticide exposure. Insect Mol Biol.

